# Lycopene Inhibits Propagation of Chlamydia Infection

**DOI:** 10.1155/2017/1478625

**Published:** 2017-08-29

**Authors:** Naylia A. Zigangirova, Elena Y. Morgunova, Elena D. Fedina, Natalia V. Shevyagina, Tatiana G. Borovaya, Vladimir G. Zhukhovitsky, Nigel H. Kyle, Ivan M. Petyaev

**Affiliations:** ^1^Gamaleya Center of Epidemiology and Microbiology, Ministry of Health, Moscow, Russia; ^2^Lycotec Ltd., Granta Park Campus, Cambridge CB21 6GP, UK

## Abstract

Chlamydiaceae is a family of obligate intracellular pathogenic bacteria with similar developmental cycles and cell biology responsible for a wide range of diseases in different hosts including genital and eye inflammatory diseases, arthritis, and inflammatory diseases of the respiratory and cardiovascular systems. In the present paper, we report that lycopene, one of the main dietary carotenoids, which is present in tomato and some other fruits, has a strong inhibitory effect on* C. trachomatis *and* C. pneumoniae *infections in alveolar macrophages. This finding was documented by both immunofluorescence analysis and electron microscopy. It was noted that lycopene treatment inhibited intracellular phase of the chlamydial developmental cycle and resulted in a significant loss of infectious progeny. The antichlamydial effect of lycopene was also confirmed in a clinical setting. There was a significant reduction of IgG antibodies against* C. pneumoniae *in the serum of volunteers treated for a month with oral ingestion of 7 mg of lycopene. Additional studies are needed to further explore the antichlamydial activity of lycopene and its possible effect on* C. pneumoniae *in relation to antichlamydial activity of lycopene to mechanisms of atherosclerosis.

## 1. Introduction

Chlamydiaceae is a family of intracellular obligate pathogenic bacteria with similar developmental cycle and cell biology responsible for wide range of diseases in different hosts including genital and eye inflammatory diseases, arthritis, and inflammatory diseases of respiratory and cardiovascular system [1, 2]. Such intracellular bacteria may also be associated with development of neurodegenerative and behavioral disorders. For example, there is evidence that* Chlamydophila pneumoniae *may promote differentiation of preadipocyte cells into mature fat cells and be possibly associated with development of metabolic syndrome, fatty liver, or nonalcoholic steatohepatitis [3].

Even though chlamydia displays many resemblances with certain Gram-negative bacteria, it is a unique phylogenetic and genetic entity distinct from other bacteria [4]. The developmental cycle of all members belonging to family* Chlamydiaceae *is remarkably similar. Intracellular infection becomes initiated with infectious but metabolically inert elementary body (EB) which differentiates within the cell inside of membrane-associated vacuole (referred to as an inclusion body) into metabolically active but noninfective reticulate (RB) body [5]. The infective cycle terminates within 48–72 hours by lysis of eukaryotic cell and release of invective progeny to neighboring cells and subsequent repeat of cellular infections in adjacent epitheliocytes [4, 5]. Whole chlamydial infectious cycle is highly dependent on host cell energy homeostasis and metabolism, since chlamydial species lack crucial enzymes for ATP biosynthesis and are defective in biosynthesis of lipids and many other organic substances [6].

High dependence of chlamydial developmental cycle from host cell metabolism creates an opportunity to control chlamydial species by modulating host cell metabolic pathway. Such alternative for antibacterial therapy becomes highly desirable due to growing number of reports about low efficacy of conventional antibiotics and antibacterial drugs in the treatment of chlamydial infection.

In the present paper, we report that lycopene induces lipid droplet formation in a cell line of alveolar macrophages, inhibits growth of chlamydiae, and reduces the level of anti-chlamydial antibodies in volunteers.

## 2. Materials and Methods

### 2.1. Reagents

Lycopene was purchased from LycoRed (London, UK) and kept in oxygen-free containers at −80°C until used in the experiments. Stock oil solutions of lycopene (15%) were prepared using olive oil and kept at −20°C. For studies in cultured cells, the 15% oil stock lycopene solution was dissolved in DMSO at concentrations of 0.75, 1.5, and 3.0 mg/ml.

Water dispersible microencapsulated lycopene was from BASF. Its 10% suspension was mixed with DMEM at final concentration of 5 mg/ml.

### 2.2. Chlamydiae Strains and Cell Lines

Strain L2/Bu434 of* C. trachomatis *and strain Kajaani 6, K6 of* C. pneumoniae *was kindly provided by Dr. P. Saikku (University of Oulu, Finland) as well as HL (human lung) cells. B10.MLM, a cell line of alveolar macrophages, was obtained from Professor A. S. Apt (Institute of Tuberculosis, Moscow, Russia). McCoy cells were obtained from the European Collection of Cell Cultures (Salisbury, UK). Cells were grown in 5% CO_2_ in DMEM supplemented with 2 mM glutamine and 10% FCS.

### 2.3. *In Vitro* Studies

*C. trachomatis *was initially propagated in McCoy cells and* C. pneumoniae *in HL cells and elementary bodies (EB) purified by Renografin gradient centrifugation as previously described [7]. Chlamydial titers were determined by infecting McCoy or HL cells with 10-fold dilutions of thawed stock suspension. Purified elementary bodies (EB) of known titer were suspended in sucrose-phosphate-glutamic acid buffer (SPG) and used as inoculums for B10.MLM cells. Cells were grown in 24-well plates until a confluence rate of 80% was reached. B10.MLM plates were infected with* C. trachomatis *or* C. pneumoniae *at multiplicity of infection (MOI) of 30 in DMEM with 5% FBS without cycloheximide and centrifuged for 0.5 hour at 1500*g*. After 1 hour of incubation at 37°C, the cell monolayers were washed with DMEM and lycopene additions were made. Oil solution of lycopene diluted with DMSO was tested at the final concentration of lycopene of 0.75, 1.5, and 3.0 *µ*g/mL in medium. Lycopene microencapsulated in dextran was added in medium up to the final concentration of lycopene of 0.125, 0.25, and 0.5 mg/ml of DMEM. Control cells received additions of solvents or microencapsulating substances (DMSO, olive oil, or cyclodextrin) as singular ingredients.

### 2.4. Immunofluorescence Staining

Infected B10.MLM monolayers grown on coverslips in 24-well plates for 24 and 42 hours were fixed with methanol.

Permeabilized cells were stained for direct immunofluorescence (IF) using FITC—conjugated species-specific monoclonal antibody against the major outer-membrane protein of* C. trachomatis *(Bio-Rad), or FITC—conjugated monoclonal antibody against chlamydial lipopolysaccharide (Nearmedic Plus, RF). Inclusion-containing cells were visualized using a Nikon Eclipse 50i fluorescence microscope at ×200 and ×1000 magnification.

### 2.5. Assessment of Infectious Progeny

For the assessment of infectious progeny, B10.MLM cells were harvested for reinfection after 42 h of cultivation, as described before [8]. Serial dilutions of lysates were inoculated onto the McCoy or HL cells. Infected cells were grown for 42 h on coverslips in 24-well plates, fixed with methanol and visualized with* C. trachomatis *species-specific or genus-specific monoclonal antibodies. The semiquantitative analysis was based on the counting of infected cells in 20 random visual fields at magnification of ×200 and calculating the mean number of inclusion forming units (IFU) per ml of the specimen. Every experiment was repeated three times.

### 2.6. Lycopene Toxicity Verification

Lycopene toxicity was controlled in MTT test (BioVision, USA) in 24 hours after lycopene addition using 96 well dishes.

### 2.7. Neutral Lipid Staining

Oil solution of lycopene diluted with DMSO was tested at the final concentration of lycopene of 3.0 *µ*g/mL in medium. Lycopene microencapsulated in dextran was added in medium up to the final concentration of lycopene of 0.5 mg/ml of DMEM. Control cells received additions of solvents or microencapsulating substances (DMSO, olive oil, or cyclodextrin) as singular ingredients. For neutral lipid staining, B10.MLM cells grown on coverslips were incubated with lycopene for 24 and 42 hours. Then cells were washed with PBS twice, fixed with 3% formaldehyde/0.025% glutaraldehyde at room temperature for 20 min, and stained with BODIPY 493/503 (Molecular Probes, Invitrogen Life Technologies, Carlsbad, CA, USA) according to manufacturer's instructions. Cells were visualized using a Nikon Eclipse 50i fluorescence microscope at ×1000 magnification.

#### 2.7.1. Automatic Image Processing Method for the Quantitative Analysis of Lipid Particles

To improve the objectivity and reproducibility of the image assessment, we developed in-house automatic immunofluorescent image processing software that allows the reception of quantitative data on intracellular lipid particles The software measures the lipid particle area in each cell from digital images of cell cultures. To perform automatic quantification we collected photos of 20 random fields of each sample. All images were uploaded into the program, and the size of lipid particle area in cells was automatically evaluated.

### 2.8. Transmission Electron Microscopy (TEM)

B10.MLM cells were cultured and infected with* C. trachomatis *with or without lycopene addition in six-well plates for a postinfection period of 42 hours and then harvested from the plates with trypsin-versene solution. Cell pellets obtained by centrifugation for 10 min at 1500 r.p.m. (Rotanta 460R; Hettich) were fixed with Ito–Karnovsky fixative solution, followed by postfixation with OsO_4_ and treatment with aqueous uranyl acetate to provide contrast. The specimens were subsequently dehydrated in an ascending series of alcohol concentrations (50, 70, 96, and 100% ethanol), infiltrated in a 1 : 1 (v/v) mixture of LR White resin and 100% ethanol for 1 h and in a pure resin for 12 h at 4°C. Resin polymerization was performed at 56°C for 24 h. Ultrathin sections were prepared, treated with a lead solution to provide contrast (Reynolds, 1963), and analysed using a JEOL 100B transmission electron microscope with an accelerating voltage of 80 kV (Jeol, Japan).

### 2.9. Statistical Analysis

All graphing and statistical analysis was conducted using ANOVA with multiple comparisons conducted relative to the cell control for statistical analysis.

## 3. Results

### 3.1. Lycopene Induces Formation of Lipid Droplets in B10.MLM Cells

**Figure 1:**
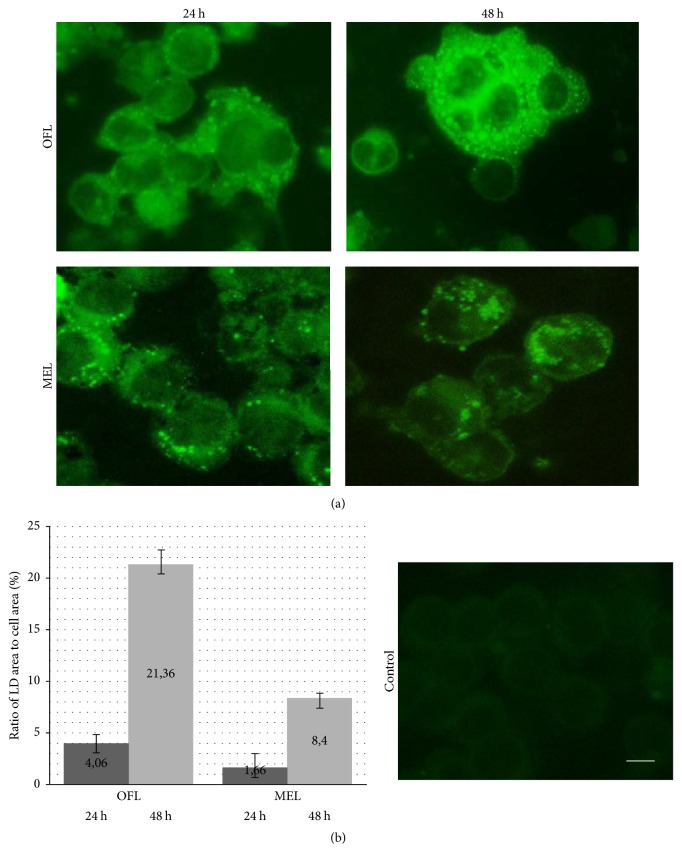
Lycopene induces formation of lipid droplets in B10.MLM cells. (a) Cells were stained with fluorescent dye BODIPY in 24 and 42 hours after lycopene addition; (b) ratio of lipid droplet area to cell area was estimated with automatic image processing method. OFL: oil-formulated lycopene and MEL: microencapsulated lycopene. Scale bar 10 *µ*M.

In 24 and 42 hours after introduction into medium of oil-formulated or microencapsulated lycopene B10.MLM cells monolayers were stained with fluorescent dye BODIPY specific for neutral lipids to evaluate lycopene lipophilic molecules intracellular storage. It was shown that inoculation of both forms of lycopene causes lipid droplet formation in B10.MLM even in 24 hours of lycopene addition to the medium (Figure 1(a)). The number of cells positive for lipid droplet formation as well as size of intracellular lipid particles was progressively increased during observation period.

To perform a reliable estimation of lycopene addition effects on lipid droplet sizes, we used in-house morphometric software based on the segmentation of cells and lipid droplets according to their different colors. The images were made at 24 and 42 hours after addition of lycopene to B10.MLM cells. The increasing ratio of lipid droplet area to cell area correlated with time of incubation (Figure 1(b)). In control dishes with olive oil and cyclodextrin, there were no lipid droplet formation.

**Figure 2:**
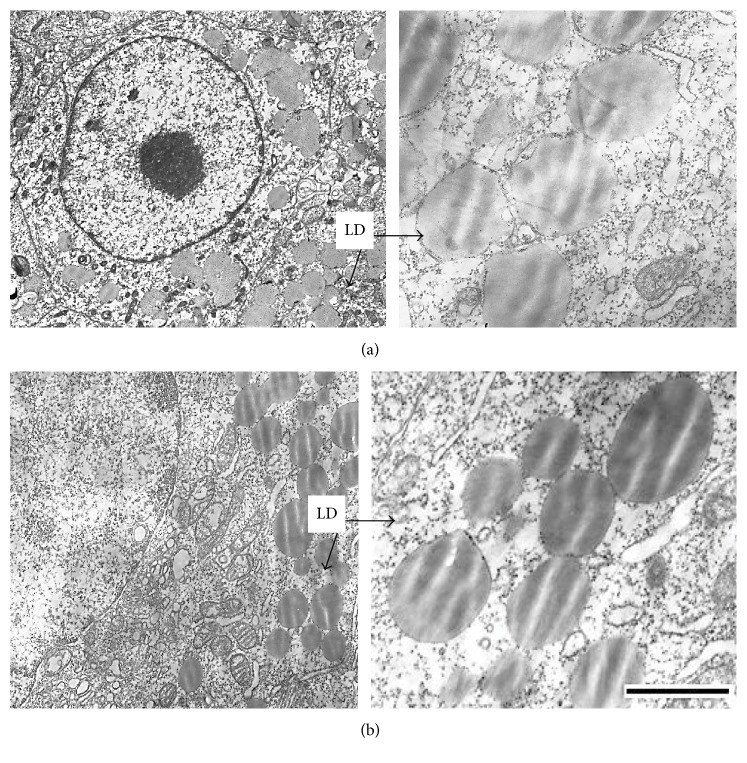
Lipid droplets in cytoplasm of B10.MLM cells. Multiple lipid droplet (LD) particles of moderate electron density in cytoplasm after incubation with oil-formulated (a) and microencapsulated (b) lycopene for 42 hours. Scale bar 1 *µ*m.

Lipid droplet formation in alveolar macrophages cell line was investigated by electron microscopy. Intact В10.МLM cells had a round-shaped form with irregular membrane surface. Cells treated with oil-formulated lycopene had the same structure like intact cells excluding appearance of lipid particles that were integrated in the membrane structure (Figure 2(a)). Lipid particles were of moderate electron density. After incubation of В10.МLM cells with microencapsulated lycopene, there were multiple numbers of lipid droplets of moderate electronic density (Figure 2(b)) without any structural changes of cell organelles.

Therefore, the obtained results suggest that incubation of cells with lycopene in oil-formulated or microencapsulated forms induced lipid droplet formation in cytoplasm without significant effect on the structure of alveolar B10.MLM cultured macrophages.

### 3.2. Lycopene Inhibits Chlamydial Infection in Dose-Dependent Manner

**Figure 3:**
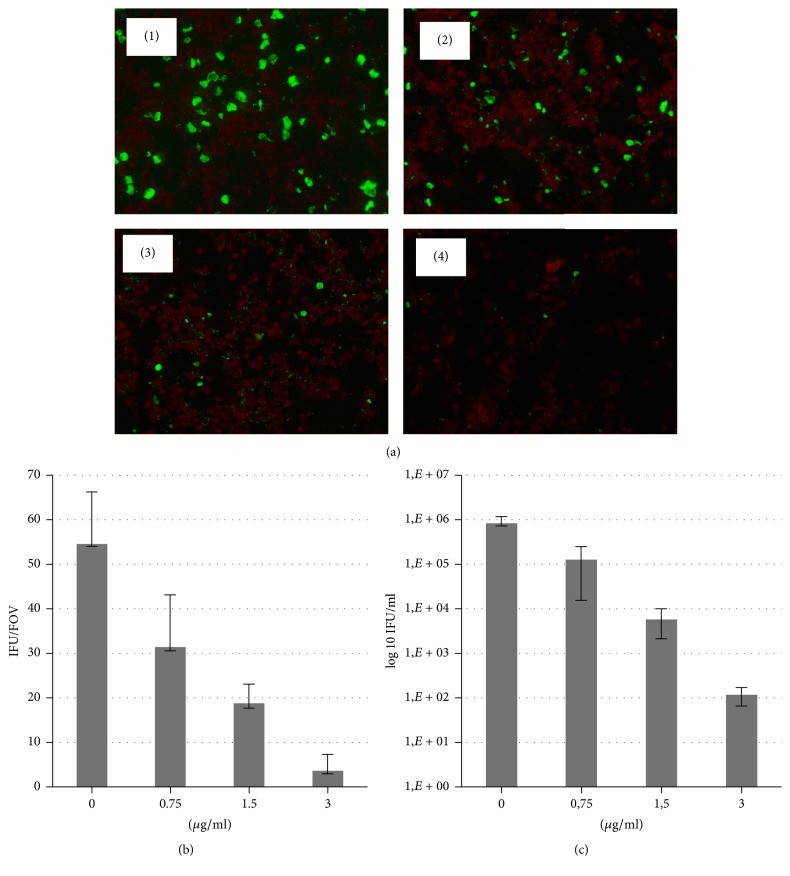
Dose-dependent inhibition of* C. trachomatis* growth in B10.MLM cells at 42 hpi in the presence of oil-formulated lycopene. (a)* C. trachomatis *infection in B10.MLM cells at 42 h.p.i. (1) growth in the presence of 0.015% olive oil in DMSO; (2) growth in the presence of 0.75 *µ*g/ml; (3) 1.5 *µ*g/ml; and (4) 3.0 *µ*g/ml of oil-formulated lycopene. Scale bar 100 *µ*m. (b) Quantitative representation of the inclusion numbers of control and lycopene treated cells. IFU/FOV = Average Inclusion Forming Units per Field of View (*n* = 20). (c) Infectious yield after treatment with different doses of lycopene.

Using immunofluorescence microscopy we monitored chlamydial inclusion size and inclusion numbers after incubation of B10M.BLM infected cells with two formulations of lycopene. As shown in Figure 3, treatment with oil-formulated lycopene caused dose-dependent blockage of* C. trachomatis *inclusion expansion. Inclusions treated with 0.75–3.0 *µ*g/ml of lycopene were smaller than in the control. In addition, the number of inclusions in the lycopene treated cultures was fewer than that of control for all tested doses with practically complete loss of inclusions at 3.0 *µ*g/ml.

**Figure 4:**
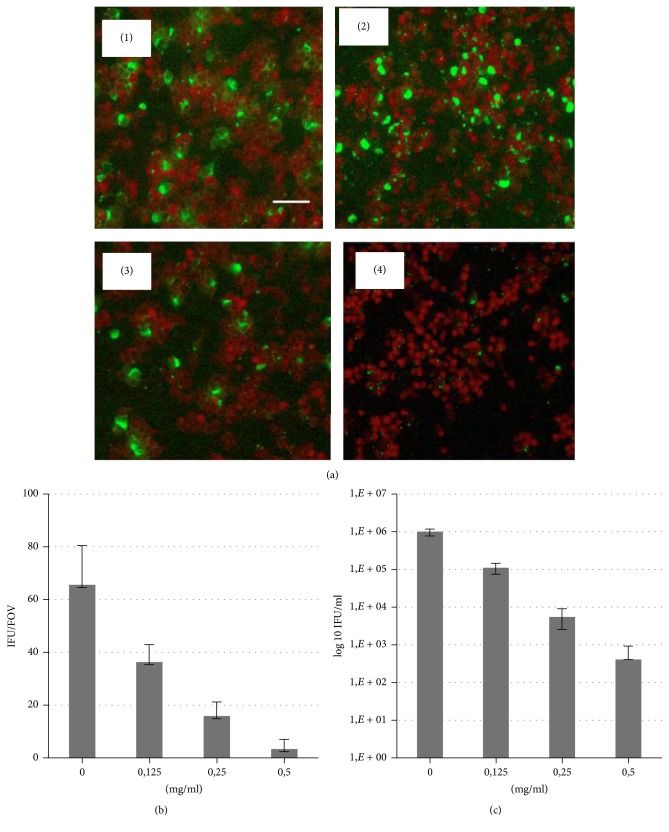
Dose-dependent inhibition of* C. trachomatis *growth in B10.MLM cells at 42 hpi in the presence of microencapsulated lycopene. (a)* C. trachomatis* infection in B10.MLM cells at 42 h.p.i. (1) growth in the presence of 1.0% cyclodextrin; (2) growth in the presence of 0.125 mg/ml; (3) 0.25 mg/ml; and (4) 0.5 mg/ml of microencapsulated lycopene. Scale bar 100 *µ*m. (b) Quantitative representation of the inclusion numbers of control and lycopene treated cells. IFU/FOV = Average Inclusion Forming Units per Field of View (*n* = 20). (c) Infectious yield after treatment with different doses of lycopene.

*C. trachomatis *infected cells treated with microencapsulated lycopene at concentrations of 0.125–0.5 mg/ml were found to have a gradual decrease in the number of infected cells and the significant reduction of inclusion bodies sizes at all tested doses (Figure 4).

It has to be noted that addition of lycopene has been performed in all cases after finalized adhesion and internalization of* C. trachomatis *by cultured cells. It excludes the possibility that inhibition of chlamydial infection observed in our studies develops due to direct effect of lycopene on bacterial pathogen and suppression of its infective abilities. There is rather influence of lycopene on replicative intracellular phase of the* Chlamydia *developmental cycle.

Infectious progeny was determined by passaging the cultures at completion of the developmental cycle after treatment. There was a significant loss of infectious progeny of* C. trachomatis *treated with both formulations of lycopene. As shown in Figures 3(c) and 4(c), lycopene treatment resulted in highly significant loss (up to 10^3-4^ log) of progeny (IFU).

Inhibition of chlamydial growth was not caused by lycopene toxicity in cell B10.MLM monolayers. Evaluation on B10.MLM cell line by using MTT assay showed that the 50% cytotoxic concentration (CC_50_) value for oil-formulated lycopene was 10.65 ± 0.3 *µ*g/mL and for microencapsulated lycopene was 8.17 ± 0.25 mg/ml indicating that both formulations are not cytotoxic.

**Figure 5:**
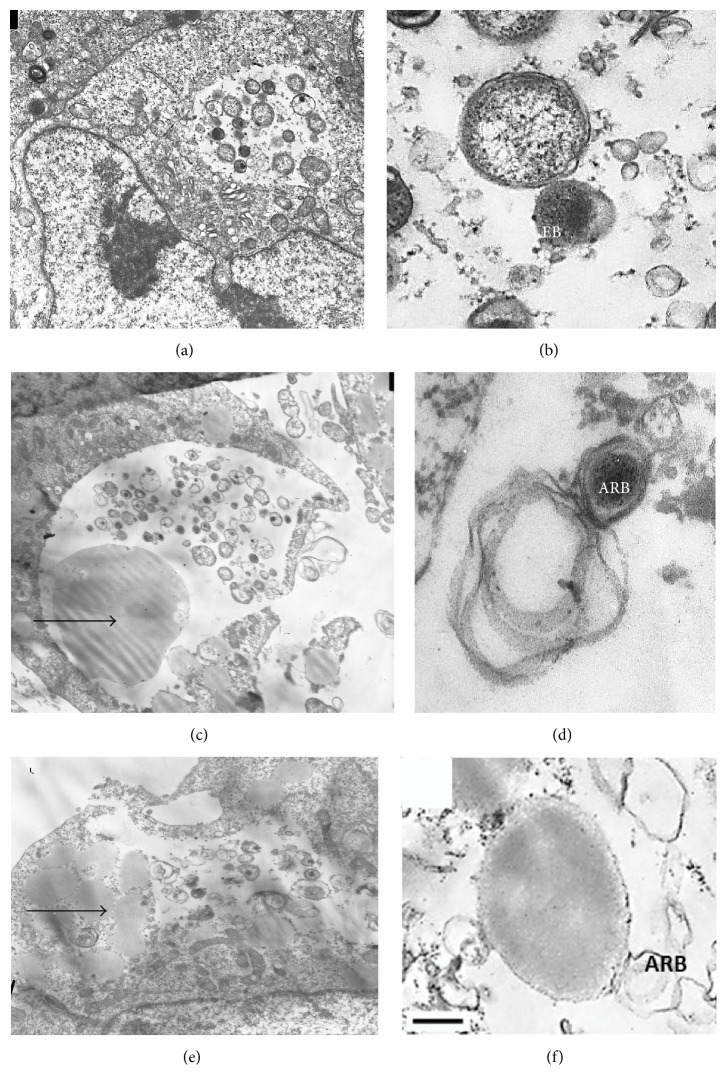
Lycopene treatment disrupts chlamydial developmental cycle in alveolar macrophages B10.MLM. ((a) and (b))* C. trachomatis *infection at 42 hpi without lycopene (EB: elementary body and RB: reticulate body); ((c) and (d))* C. trachomatis *infection at 42 hpi treated with oil-formulated lycopene (ARB: abnormal reticulate body); ((e) and (f))* C. trachomatis* infection at 42 hpi treated with microencapsulated lycopene. Lipid droplets are in close contact to and inside chlamydial inclusions. Arrows indicate lipid droplets. Bar = 0.25 *µ*m.

Using transmission electron microscopy, it was shown that* C. trachomatis *infected В10.МLM cells had unchanged shape. There were multiple vacuoles containing chlamydial inclusion bodies at different stages of life cycle (Figures 5(a) and 5(b)) with typical elementary and reticulate bodies.

After incubation of infected В10.МLM cells with 3 *µ*g/ml oil-formulated lycopene for 42 hours, multiple lipid particles were found to be located in cytoplasm and even in some chlamydial inclusion bodies, which were detected rarely.* C. trachomatis *reticulate bodies had abnormal morphology with expanded periplasmic space or disrupted structure (Figures 5(c) and 5(d)).

After incubation of infected В10.МLM cells with microencapsulated lycopene (0.5 mg/ml), there were enlarged and disrupted inclusions with singular chlamydial atypical reticulate bodies, whereas elementary bodies were absent. Lipid droplets were located in cytoplasm and in close contact with* C. trachomatis *inclusions in infected В10.МLM cells (Figures 5(e) and 5(f)).

**Figure 6:**
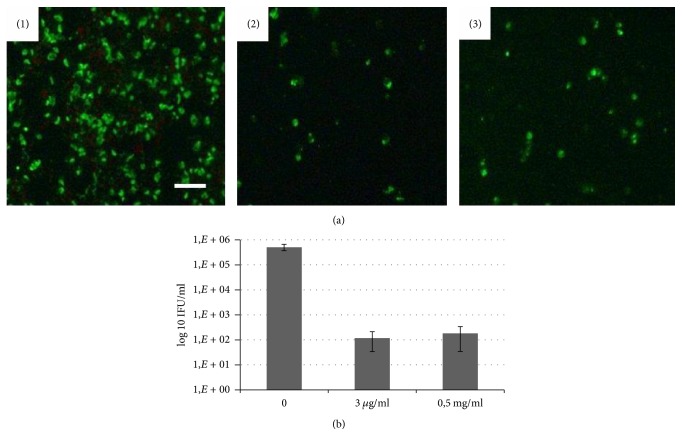
Inhibition of* C. pneumoniae *growth in B10.MLM cells in the presence of two formulations of lycopene. (a)* C. pneumoniae *infection in B10.MLM cells at 72 h.p.i. (1) growth without lycopene; (2) 3.0 *µ*g/ml of oil-formulated lycopene; and (3) 0.5 mg/ml of microencapsulated lycopene. Scale bar 100 *µ*m. (b) Infectious yield after treatment with two formulations of lycopene.

In another set of experiments it was demonstrated that lycopene could also inhibit growth and propagation of* Chlamydophila pneumoniae *infection in cell culture (Figure 6). Treatment of* C. pneumoniae *infected B10.MLM cells with oil-formulated lycopene (0.3 *µ*g/ml) or microencapsulated lycopene (0.5 mg/ml) induced loss of inclusions from the cultures and significantly reduced the infectious progeny (Figure 6(b)).

### 3.3. Lycopene Reduces Antichlamydial Antibody Titer in Cardiovascular Patients

**Figure 7:**
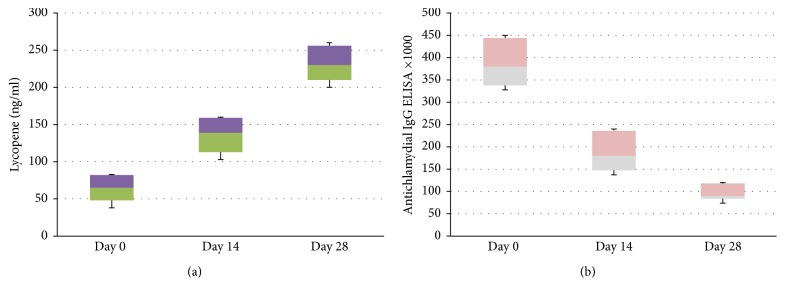
Changes in serum lycopene level (a) and serum chlamydia-specific IgG antibodies (b) in volunteers treated with 7 mg of GA lycopene for 28 days.

To confirm the effect of lycopene on chlamydial infection in clinical settings a pilot clinical study was conducted. 36 patients with cardiovascular disease and positive for the presence in their blood of IgG anti-chlamydial pneumoniae antibodies (as verified by Medac ELISA Test, Hamburg, Germany) were enrolled and treated with a single daily oral dose of proprietary GA lycopene containing 7 mg of active substance for 4 weeks (Lycotec Ltd., Cambridge, UK). First of all, it was found that such a regimen of lycopene intake leads to a stable increase of lycopene level in serum as compared to the pretreatment values (Figure 7(a)). Secondly, there was a stepwise reduction of antichlamydial IgG in the serum of cardiovascular patients three times below baseline level (Figure 7(b)).

## 4. Discussion

According to recent statistics from the Center of Disease Control and Prevention (CDC), chlamydiosis is the most common reportable disease in the United States. Two chlamydial pathogens have the most notable impact on human health:* C. trachomatis *and* C. pneumoniae*.* C. trachomatis *infects over 100 million of people worldwide.* C. pneumoniae *is the most commonly occurring intracellular bacterial pathogen in respiratory system; it is of unknown worldwide prevalence and has more than 50% prevalence in people with cardiovascular pathologies or risk of their development [9–14]. There are worrisome reports about drug resistance of chlamydial pathogens to the commonly used antibiotics via lateral and horizontal mutated gene transfer [15]. It is of clinical importance that, under the selective pressure of beta-lactam antibiotics, interferon- gamma, or deprivation of nutrients such as iron and amino acids (e.g., tryptophan), most of chlamydiae can enter a persistent, metabolically inactive state that is refractory to current antibiotic treatment strategy. That is why the search of new approaches to the treatment of chlamydial infections remains important. There are a small number of new drugs currently in preclinical development and early clinical trials that may have a role in the treatment of chlamydial infections.

Nutritional factors play an extraordinary role in the mechanisms of antiviral and antibacterial resistance. The recent body of evidence demonstrates that some carotenoids display a high range of antibacterial activity towards various microorganisms [16]. It has also been recently discovered that propagation of* Chlamydiae *might be affected by phytochemicals. In particular, luteolin prevents acute* C. pneumoniae *infection in mice and reduces inflammation in the lung tissue [17].

In the present paper, we report that lycopene, one of the main dietary carotenoids, which is present in tomato and some other fruits, has a strong inhibitory effect on* C. trachomatis *and* C. pneumoniae *infections in alveolar macrophages. This finding was documented in our studies by both the immunofluorescence analysis and electron microscopy. It has to be noted that degree of lycopene inhibitory of both chlamydia growth was overwhelming and reached over 90% according to the immunofluorescence analysis. The antichlamydial effect of lycopene was also confirmed in a clinical setting. There was a significant reduction of IgG antibodies against* C. pneumoniae *in the serum of volunteers treated for a month with oral ingestion of 7 mg of GA lycopene (Lycotec Ltd., Cambridge, UK).

It is essential to mention again that the study protocol excludes any possibility of direct effect of lycopene on viability and/or infectivity of* C trachomatis *and* C pneumoniae *during cell exposure to the pathogen since addition of lycopene was performed in the postattachment period of chlamydial infection when infective particles were washed out from the dishes. Therefore, the inhibitory effect of lycopene on chlamydial growth develops according to our results solely due to the effect of lycopene on intracellular events accompanying propagation of* C trachomatis *and C* pneumoniae *in the host cells.

There are various possible mechanisms for the inhibitory effect of lycopene on chlamydia infection in cultured cells. First of all, as we reported above, incubation of cultured cells with lycopene leads to accumulation of lipid droplets in cultured cells. The chlamydial life cycle is highly dependent on host cell metabolism. In particular,* Chlamydiae *cannot synthesize lipids and must acquire lipids from the host cells. It might be a possibility that lipid overloading of host cells as well as lipid deficiency in cultured cells are not favorable for intracellular growth of chlamydial pathogen. This assumption is in good agreement with our previous results revealing the effect of HMG-CoA reductase inhibitors on* C. trachomatis *infection as well as other reports suggesting antichlamydial effect of inhibitors of cholesterol biosynthesis [8, 18]. Moreover, lycopene is a well-known inhibitor of HMG-CoA reductase, a rate-limiting enzyme of cholesterol biosynthesis. Therefore, inhibition of cholesterol biosynthesis in cultured cells infected with* C trachomatis *might be a key mechanism of attenuation of chlamydial replication in host cells. Such an assumption is well supported by the recently published results revealing the inhibitory effect of statins on chlamydial infection [19, 20].

However, the most important conclusion comes from the fact that lycopene also has an ability to block infection caused by* C. pneumoniae. *According to our results, the inhibitory effect of lycopene on chlamydial infection is almost the same in cells infected with either chlamydial pathogens:* C. trachomatis or C. pneumoniae*. Moreover, according to our results, the lycopene treatment also reduces the titer of* C. pneumoniae-*specific antibodies in volunteers. Rapid decline in the chlamydia-specific IgG observed in our work needs to be addressed in further studies. If lycopene possesses antichlamydial* in vivo* conditions, chlamydia-specific IgG can bind chlamydial particles and their remnants released from ruptured cells and undergo rapid receptor-mediated clearance from the blood stream by the cells of reticuloendothelial system. Such an assumption becomes a possibility due to our previous results describing identification of* C. pneumoniae* in serum specimens of patients with cardiovascular disease [21].

As widely believed [22],* C. pneumoniae *is a bacterial pathogen deeply implicated in the initiation, pathogenesis, and resolution of atherosclerosis. Therefore, in our opinion, the antichlamydial effects of lycopene may explain multiple pieces of epidemiological evidence [23] suggesting a favorable effect of the Mediterranean diet on cardiovascular health and directly link lycopene ingestion and decreased risk of cardiovascular disease [24, 25]. It is very likely that ingestion of lycopene by individuals adhering to the Mediterranean diet confers upon them a certain degree of protection from cardiovascular disease [26].

And finally, the inhibitory effect of lycopene on chlamydial infection might be directly related to the antioxidant properties of lycopene. As recently shown, the initial phase of chlamydial infection is accompanied by a significant spike in production of reactive oxygen species [27]. Subsequently, antioxidant interventions are capable of blocking chlamydial replication in host cells [28]. Additional studies are needed to further explore the antichlamydial activity of lycopene and its possible effect on chlamydia-mediated mechanisms of atherosclerosis.
